# Ruptured Rudimentary Horn Pregnancy at 33 Weeks of Gestation With a Live Baby

**DOI:** 10.7759/cureus.114660

**Published:** 2026-08-17

**Authors:** Ashish Singh, Sonia Naik, Heera P, Tanya Rohatgi, Binita Neupane

**Affiliations:** 1 Obstetrics and Gynecology, Max Super Speciality Hospital, Lucknow, IND; 2 Obstetrics and Gynecology, Max Super Speciality Hospital, Delhi, IND; 3 Obstetrics and Gynecology, Max Smart Super Speciality Hospital, Delhi, IND; 4 Reproductive Medicine, Max Super Speciality Hospital, Delhi, IND; 5 Obstetrics and Gynecology, Max Smart Superspecilaity Hospital, Delhi, IND

**Keywords:** antepartum hemorrhage, emergency caesarean section, emergency obstetric care, preterm delivery, ruptured rudimentary horn pregnancy, emergency laparotomy

## Abstract

Pregnancy in a complex Müllerian anomaly is an uncommon and potentially catastrophic obstetric condition. It often remains undiagnosed until the patient presents in an emergency setting. Presentation of a rudimentary horn pregnancy near term is uncommon, as most patients present earlier with acute abdominal pain and/or features of hypovolaemic shock following rupture.

The diagnosis of a ruptured rudimentary horn pregnancy at the time of presentation is challenging and may be misinterpreted as other obstetric emergencies, such as uterine rupture, placental abruption, or bleeding from placenta percreta. Delayed diagnosis and inadequate intervention can significantly increase maternal and neonatal morbidity and mortality.

We successfully salvaged the mother and fetus in a case of complex Müllerian horn pregnancy that presented with acute abdominal pain and features of hypovolaemic shock. A high index of suspicion for uterine rupture was maintained until surgery.

## Introduction

Müllerian duct anomalies are associated with congenital abnormalities of the renal system, with a reported prevalence of 30%-50%, and vertebral anomalies occurring in approximately 29% of cases [1]. The prevalence of Müllerian anomalies among women with infertility and recurrent miscarriage is approximately 8% and 13.3%-24.5%, respectively [2].

Patients may present with lower abdominal pain, primary amenorrhoea, infertility, and obstetric complications such as ectopic pregnancy, miscarriage, preterm labor, and malpresentation [3]. Patients with a functional non-communicating rudimentary horn may present with severe or progressive cyclical dysmenorrhoea and lower abdominal or pelvic pain due to obstruction of menstrual outflow, which may result in haematometra and, in some cases, endometriosis. In contrast, a communicating rudimentary horn generally permits menstrual drainage and may therefore cause fewer obstructive menstrual symptoms, allowing the anomaly to remain undiagnosed until pregnancy or an obstetric complication [4].

Ultrasonography remains the first-line imaging modality, while MRI provides superior anatomical delineation; however, diagnostic accuracy remains limited, and many cases are diagnosed intraoperatively [5].

Pregnancy in a rudimentary horn is rare, with an estimated incidence ranging from 1 in 75,000 to 1 in 150,000 pregnancies [6]. The fertilized ovum usually reaches the rudimentary horn through transperitoneal migration. Owing to the poor musculature and limited distensibility of the horn, there is a significant risk of rupture [7]. Approximately 80%-90% of rudimentary horn pregnancies rupture by the second trimester, and only 14% are diagnosed before the onset of symptoms [8].

Here, we report a case of a ruptured rudimentary uterine horn pregnancy presenting at 33 weeks of gestation with a live baby. This case illustrates the diagnostic challenge posed by this rare presentation. It underscores the importance of a high index of suspicion and prompt surgical intervention in achieving a favorable maternal and neonatal outcome.

## Case presentation

A woman in her 20s, an unbooked primigravida at 33 weeks' gestation, presented to the emergency obstetric department with severe abdominal pain, features of shock, and reduced fetal movements for seven to eight hours.

On examination, she appeared anxious and distressed. Her pulse rate was 120 beats per minute, blood pressure was 99/58 mm Hg (progressively falling), and oxygen saturation was 98% on room air. Abdominal examination revealed a term-sized uterus with generalized rebound tenderness. Vaginal examination showed a closed, uneffaced cervix with no bleeding.

Due to severe pain, adequate ultrasound assessment was difficult. A fetal heart rate of 122-135 beats per minute was noted, along with a large amount of clot. The fetal heart was also confirmed using a stethoscope near the umbilicus.

The patient was taken for emergency laparotomy with a provisional diagnosis of uterine rupture, placental abruption, or, less likely, bleeding from placenta percreta.

On opening the abdomen, approximately 1500-2000 mL of blood mixed with amniotic fluid and clots was encountered. The fetus was found in the upper abdomen and delivered in breech presentation. The neonate had a weak cry at birth. Birth weight of the neonate (male) was 1.85 kg.

**Figure 1:**
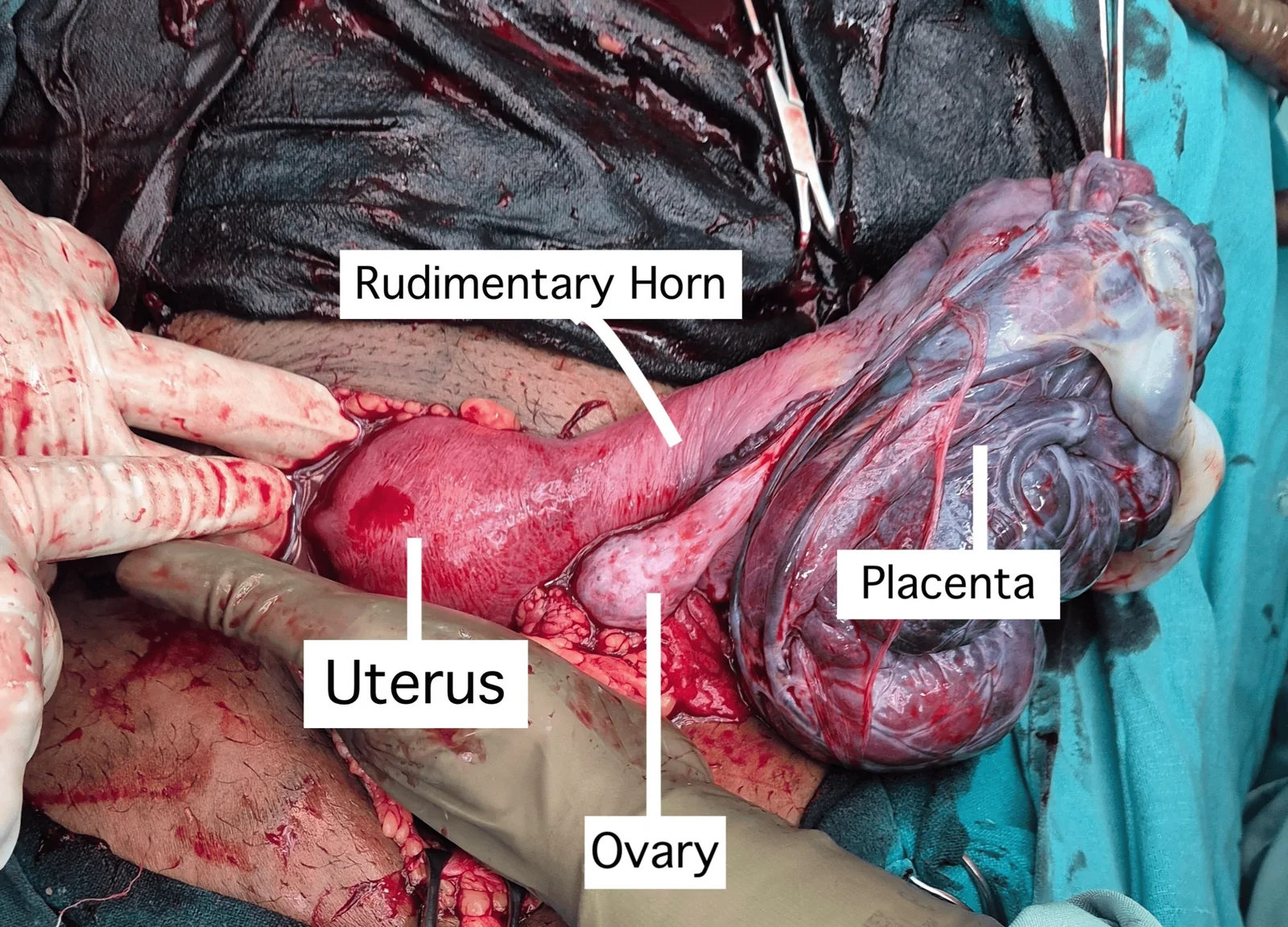
Anatomical demonstration of a ruptured rudimentary horn pregnancy Intraoperative image showing a ruptured non-communicating rudimentary uterine horn following delivery of a live fetus at 33 weeks' gestation. The unicornuate uterus is visualised separately from the ruptured rudimentary horn containing the placenta. The ipsilateral ovary is preserved. Labels identify the uterus, rudimentary horn, placenta, and ovary to demonstrate the operative anatomy.

The uterus was approximately 12-14 weeks in size. After achieving hemostasis, a rudimentary horn was identified on the right side, with the placenta attached to it. The pregnancy was located entirely outside the uterine cavity. The rudimentary horn was excised along with a right salpingectomy, and the uterus was repaired (Figure 1).

Both ovaries were normal, and no additional anomalies were identified. Two units of packed red blood cells were transfused intraoperatively, and the patient was shifted to the intensive care unit.

Further history revealed no prior antenatal care. She reported a brief episode of abdominal pain two weeks earlier, which resolved spontaneously; it is difficult to attribute this episode definitively to the rudimentary horn, as it was not associated with any other complaint, and the pregnancy subsequently remained viable.

Investigations

Routine laboratory investigations, together with the maternal vital signs recorded at presentation and the neonatal umbilical artery pH, are summarised in Table 1. Coagulation profile and renal and liver function tests were within normal limits.

**Table 1:** Maternal vital signs, laboratory investigations, and neonatal umbilical artery pH *Coagulation profile, renal function test, and liver function test were reported as within normal institutional limits in the case notes; individual numerical values were not recorded and so cannot be reproduced here.

Parameter	Patient value	Unit	Reference range
Maternal vital signs (at presentation)			
Pulse rate	120	bpm	60-100
Blood pressure (systolic/diastolic)	99/58, progressively falling	mm Hg	90/60-120/80
Oxygen saturation (SpO₂)	98	% (room air)	94-100
Fetal heart rate (FHR)	122-135	bpm	110-160
Maternal laboratory investigations			
Haemoglobin (Hb)	9	g/dL	12-16
Platelet count	1.6	lakh/µL	1.5-4.0
Total leucocyte count (TLC)	9000	/µL	4000-11,000
Coagulation profile (PT/aPTT/INR)	Within normal limits*	-	Normal
Renal function test (RFT)	Within normal limits*	-	Normal
Liver function test (LFT)	Within normal limits*	-	Normal
Neonatal parameter			
Umbilical artery pH	7.17	-	7.20-7.30

Outcome and follow-up

The mother was monitored in a high-dependency unit for 24 hours. The neonate had Apgar scores of 5, 7, and 9 at 5, 7, and 10 minutes, respectively, and was admitted for observation. Positive pressure ventilation support was given to the baby for 24 hours. Breastfeeding was initiated on day three. Umbilical artery pH was 7.17 (reference range 7.20-7.30) (Table 1).

Both mother and baby were discharged on postoperative day seven. At one-year follow-up, the child demonstrated normal developmental milestones.

Treatment

During hospitalization, the patient was administered intravenous antibiotics, including cefuroxime 1.5 g twice daily for three days and metronidazole 500 mg three times daily for one day. She received intravenous pantoprazole 40 mg twice daily for three days. Analgesia was provided with intravenous paracetamol 1 g four times daily, alternating with intravenous diclofenac 75 mg four times daily for three days. Antiemetic therapy included intravenous metoclopramide 10 mg twice daily for three days. Intravenous fluids were administered as per clinical requirements.

## Discussion

Continuation of a complex Müllerian horn pregnancy to near term is rare and is infrequently reported in the literature [9,10]. The factor responsible for the continuation of pregnancy in a rudimentary horn is thought to be highly dependent on the thickness of its musculature, the distensibility of the rudimentary horn, its vascular supply, and the presence of communication with the main uterine cavity. The available literature suggests that variation in these anatomical factors may contribute to the wide variation in gestational age at rupture and, in rare cases, may allow pregnancy to progress into the third trimester [7,10]. Further studies are required to assess the relationship between horn thickness and the continuation of pregnancy.

Another notable point in this case is the presence of a communicating rudimentary horn with otherwise normal uterine anatomy. According to the classification of the American Society for Reproductive Medicine, this may be considered a complex Müllerian anomaly [2]. However, classification remains difficult, as the rest of the reproductive anatomy was normal.

Early recognition and prompt surgical management are essential to reduce maternal and neonatal morbidity and mortality [8,11]. A detailed, methodical evaluation is not always possible in this setting, as the delay may result in the loss of the patient or the baby; in this case, timely presentation and prompt decision-making allowed both lives to be saved. A high index of suspicion in patients presenting with acute abdomen and shock during pregnancy, especially in unbooked cases, remains a challenge, and the final diagnosis is often made on the operating table [5,9].

Maternal and neonatal health remain high priorities in all countries, and access to comprehensive emergency obstetric care at a tertiary center is associated with reduced maternal and neonatal morbidity and mortality [11]. This case had a successful outcome due in part to presentation at a tertiary care hospital with immediate access to laparotomy, blood transfusion, and neonatal resuscitation facilities. Had the patient presented to a center without these facilities, the outcome may have been different, although this cannot be established from a single case.

## Conclusions

Detailed evaluation is not always possible in obstetric emergency cases, which require prompt action and a multidisciplinary approach to optimise maternal and fetal outcomes. In such acute situations, the definitive diagnosis may only become apparent intraoperatively, highlighting the need for clinical vigilance and flexibility in management.

A communicating rudimentary horn with an otherwise normal uterus does not fit neatly into standard Müllerian anomaly classifications; intraoperative confirmation of the horn's architecture and its relationship to the uterine cavity is essential, as this directly determines the surgical approach- in this case, excision of the horn with salpingectomy rather than a more conservative option.
